# L-arginine supplementation and risk factors of cardiovascular diseases in healthy men: a double-blind randomized clinical trial

**DOI:** 10.12688/f1000research.5877.2

**Published:** 2017-06-22

**Authors:** Naseh Pahlavani, Mostafa Jafari, Omid Sadeghi, Masoud Rezaei, Hamid Rasad, Hossein Ali Rahdar, Mohammad Hasan Entezari

**Affiliations:** 1 Department of Nutrition, Faculty of Medicine, Mashhad University of Medical Sciences, Mashhad, Iran; 2 Food Security Research Center and Department of Clinical Nutrition, School of Nutrition and Food Science, Isfahan University of Medical Sciences, Isfahan, Iran; 3 Student Research Committee, Arak University of Medical Science, Arak, Iran; 4 Department of Community Nutrition, School of Nutritional Sciences and Dietetics, Tehran University of Medical Sciences, Tehran, Iran; 5 Faculty of Nursing and Midwifery, School of Medicine, Isfahan University of Medical Sciences, Isfahan, Iran; 6 Department of Microbiology, School of Medicine, Isfahan University of Medical Sciences, Isfahan, Iran

## Abstract

**Context: **The effect of L-arginine on risk factors of cardiovascular diseases (CVD) has mostly focused on western countries. Since cardiovascular diseases is the second cause of death in Iran and, as far as we are aware, there have been no studies about the effect of L-arginine on CVD risk factors, the aim of this trial was to assess the effects of L-arginine supplementation on CVD risk factors in healthy men.

**Objective:** The purpose of this study was to evaluate the effect of low-dose L-arginine supplementation on CVD risk factors (lipid profile, blood sugar and blood pressure) in Iranian healthy men.

**Design, setting, participants:** We conducted a double-blind randomized controlled trial in 56 patients selected from sport clubs at the Isfahan University of Medical Science between November 2013 and December 2013.

**Interventions: **Healthy men received L-arginine supplementation (2000 mg daily) in the intervention group or placebo (2000 mg maltodextrin daily) in the control group for 45 days.

**Main outcome measure:** The primary outcome measures were we measured the levels of fasting blood sugar, blood pressure and lipid profile including triglyceride (TG), cholesterol, LDL and HDL in healthy subjects. It was hypothesized that these measures would be significantly improved in those receiving L–arginine supplementation. at the beginning and end of the study.

**Results:** In this trial, we had complete data for 52 healthy participants with mean age of 20.85±4.29 years. At the end of study, fasting blood sugar (P=0.001) and lipid profile (triglycerideTG (P<0.001), cholesterol (P<0.001), LDL (P=0.04), HDL (P=0.015)) decreased in the L-arginine group but we found no significant change in the placebo group. In addition, the reduction of fasting blood sugar and lipid profile in L-arginine was significant compared with placebo group. No significant changes were found about systolic (P=0.81) and diastolic blood pressure either in L-arginine or placebo group. (P=0.532).

**Conclusion**: The use of L-arginine significantly improved outcomes compared to placebo.

## Introduction

High blood pressure is now considered one of the main challenges facing human health and is one of the most important risk factors for cardiovascular disease ^1^. It is predicted that the incidence of cardiovascular disease and hypertension will reach in about 30% of the world population by the year 2025. Iran is the fifth country in the world in terms of having high blood pressure related diseases. Approximately 6.6 million with an age range of 25–64 years have high blood pressure and an estimated 12 million people in the same age range are at increased risk of hypertension and cardiovascular disease ^2, 3^. One of the major mechanisms of cardiovascular disease is endothelial dysfunction. Dysfunction, which can increase the permeability of the plasma components, especially low-density lipoproteins (LDL) and deposition in the sub endothelial space, can be considered one of the earliest events that occur in atherosclerosis ^4, 5^. With the high prevalence of hypertension and cardiovascular diseases and complications, and high costs that they impose on society, presentation of new strategies for prevention and control of these diseases, as well as finding efficient and effective complementary therapies with few instances of complications is very important ^6^.

L-arginine is a semi-essential amino acid that is used by all cells ^7^. This amino acid, on average, constitutes 7–5% of the total amino acids in the normal human diet and is absorbed in the jejunum and ileum of the small intestine. L-arginine is used by the body in protein synthesis, urea cycle, tissue repairing and immune cell function ^8, 9^. Arginine is converted to nitric oxide and citrulline, which acts as a vasodilator. There are three isoforms of nitric oxide synthase (NOS), these isoforms need oxygen, arginine and Tetrahydrobiopterin 4 (BH4) and NADPH (nicotine amide adenine di nucleotides phosphate) for the synthesis of nitric oxide ^10^. In a trial conducted on Zucker rats, L-arginine reduced adipose tissue ^11^.

Recently, arginine-rich foods were shown to be inversely associated with endothelial dysfunction in hypercholesterolemia patients ^12^. It has also been shown that long-term administration of L-arginine reduces cardiovascular complications ^13^. It is still not entirely clear that a low dose of L-arginine has a positive effect. Nitric oxide has an important function in fat metabolism ^14^. Physiological levels of nitric oxide (25 to 35 µmol) increased oxidation of glucose and fat and prevented the synthesis of glucose and triglycerides ^15^. Several amino acids, particularly arginine, glutamine, leucine and phenylalanine directly stimulate the production of insulin from pancreatic beta cells ^16^. Other possible actions associated with L-arginine include lowering blood pressure and homocysteine levels, increasing lean body mass and decreasing fat mass and adiponectin and endothelin ^17^. In one study conducted by Sato *et al.*, infusion of L-arginine reduced blood pressure in patients with essential hypertension but was not effective in patients with a history of dangerously high blood pressure ^18^.

In a study conducted on healthy volunteers, supplementing with L-arginine for 3 days in a week improved glucose metabolism ^19, 20^. In a study, Lucotti *et al.* demonstrated that prolonged treatment with L-arginine in patients with type 2 diabetes caused a significant decrease in blood sugar ^21^. In general, a number of studies on the beneficial effects of L-arginine have been shown to reduce blood pressure ^22^. But in some other studies, L-arginine had no effect on blood pressure ^23, 24^. In previous studies, the effects of long-term, low L-arginine intake have not been examined.

To best of our knowledge, no study has evaluated the effects of 2g of L-arginine per day on cardiovascular risk factors. Based on the L-arginine dosage used in Elam *et al.* ^25^, and based on their conclusion that increasing the duration of L-arginine supplementation can be more effective than increasing the dosage of L-arginine, this clinical trial aimed to evaluate the effects of 45 days L-arginine supplementation (2g per day) on lipid profiles, fasting blood glucose and blood pressure in male athletes. In the present study, we hypothesized that 2g per day L-arginine supplementation during 45 days provides better outcomes on cardiovascular risk factors than other dosages used in previous studies.

Therefore, in this study, we examined the effect of L-arginine supplementation on lipid profile, blood pressure and fasting blood sugar (glucose; FBS) in healthy men.

## Material and methods

### Study design

This double-blind randomized clinical trial (IRCT2013060411763N9) was conducted on 56 healthy male sports club members of Isfahan University of Medical Sciences, Isfahan, Iran, from November 2013 to December, 2013.

### Inclusion and exclusion criteria

Male participants, with no history of smoking or alcohol consumption during the past year, not taking nutritional sport supplements during the last 2 months, no acute or chronic illness (including mental disorders, untreated hypothyroidism, heart and kidney disease, hepatitis, infectious and inflammatory diseases) and 18 to 35 years of age were included in this trial. Participants with any of the aforementioned diseases were excluded from this study.

### Sample size

Participants were invited to participate in the study by advertising at sports clubs at Isfahan University of Medical Sciences, Isfahan, Iran. A total of 70 men participated in the study, 56 subjects of which fitted the inclusion criteria. The required sample size was determined by following formula, considering a study power of 80%, a type I error of 5% (α = 0.05) and type II error of 20% (β = 0.20).

$$\text{N}\;\text{=}\;\frac{2(\text{Z}1\;+\;Z2)\sigma^{2}}{d^{2}}$$

### Data collection

We held five meeting with participants. In the first meeting, we obtained basic information using a general questionnaire. For dietary assessment, three-day dietary records of subjects (sessions 2, 3 and 4) were completed and the nutrient content of foods were determined by the Nutritionist 4 software (version 7.0; N-Squared Computing, Salem, OR), which was designed for evaluation of Iranian foods. Participants were instructed to record, as accurately as possible, everything they consumed during the day including supplements and between-meal and late-evening snacks. Physical activity level was assessed at baseline (session 1) and at the end of study (session 5) by using the IPAQ questionnaire, which is both a reliable (Tang K Hong *et al.*) and valid (Coral *et al.*) measure ^26, 27^. Weight was measured without shoes while the participants wore underwear and were recorded to the nearest 0.5 kg. BMI was calculated as weight in kilograms divided by height in meters squared. Height was measured without shoes while the shoulders were in a normal position.

Fasting blood samples were collected at day 0 (session 1) and day 45 (session 5) of this trial. The blood samples were separated at 4°C for 10 min centrifugation at 4000 rpm and the serum was frozen at -80°C until analysis. FBS levels and lipid profile including total cholesterol (TC), triglyceride (TG), LDL and HDL, were measured using Auto Analyser Biosystems A25 (BioSystems S.A., Barcelona, Spain). Blood pressure was measured three times in every session after a 15 minute rest sitting down by mercury sphygmomanometer and the average blood pressure obtained was recorded at each stage.

### Intervention

After obtaining informed consent and with the approval of the ethics committee of Isfahan University of Medical Sciences, 56 healthy men participated in this study and were randomly assigned to consume L-arginine supplement ( *n* = 28) or placebo ( *n* = 28) for 45 days using envelopes containing numbers from a table of random numbers. Pure L-arginine supplements and placebo (maltodextrin) were purchased from a pharmaceutical company (Karen Pharmaceutical Co, Yazd-Iran). Participants were instructed to take one table per day (2000 mg of L-arginine in the L-arginine group, 2000 mg of maltodextrin in the placebo group). When the participants were given packets of L-arginine or placebo they were asked not to change the lifestyle, physical activity and diet during the study. For blinding, L-arginine and placebo packets were coded by someone outside the research team and the research team was unaware of the type of supplement. The L-arginine and placebo packets were delivered to participants at session 1 and 3. They were asked to bring back the empty packets at session 3 and the final session. The statistician was also not aware of the type of intervention. At the end of the project the final report determined the type of intervention. This trial was approved by the Isfahan University of Medical Sciences (with the number 392435) and was registered in clinical trials center’s website address ( www.irct.ir) (code: IRCT2013121515807N1).

### Adverse effects

The incidence of adverse events was evaluated by recording all observed or volunteered adverse events. For this purpose, any study related adverse events during intervention were monitored by daily evaluation. For participants who withdrew or subjects lost to follow-up, adverse events were acquired by telephone.

### Statistical analysis

All statistical analyses were done by means of SPSS software version 18 (SPSS, Inc. Chicago, IL, USA). We applied Kolmogrov–Smirnov test to ensure the normal distribution of variables. To determine the differences in general characteristics and dietary intakes between L-arginine and placebo groups, we used an independent-samples t-test. We used paired-samples t-tests to determine the effects of L-arginine and placebo on FBS, lipid profile and blood pressure. P-value < 0.05 was considered as the level of significance.

## Results

Clinical data on the effect of L-arginine supplementation on cardiovascular risk factors in healthy men Data were collected from 52 healthy male sports club members (aged 18-35) at the Isfahan University Medical Sciences, Ishfan Iran. This was a double blind randomized controlled study where the intervention group were given 2000 mg L-arginine per day and the placebo group were given 2000mg maltodextrin per day over 45 days (November-December 2013). Please see associated article for more detailed methods. Click here for additional data file. Copyright: © 2017 Pahlavani N et al. 2017 Data associated with the article are available under the terms of the Creative Commons Zero "No rights reserved" data waiver (CC0 1.0 Public domain dedication).

**Figure 1.**
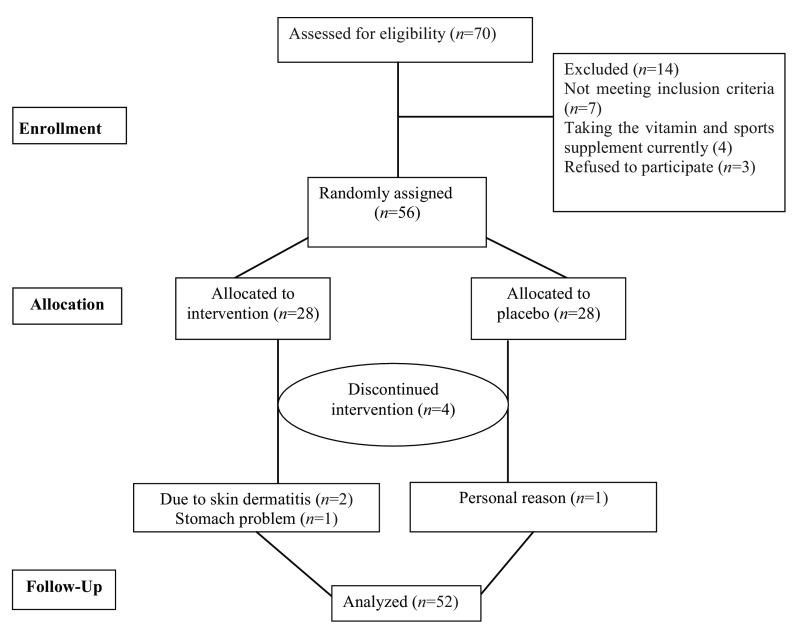
Schematic diagram of the study; Individuals in the L-arginine group received capsule containing 2000 mg of L-arginine per day during the study; those in the placebo group received 2000 mg placebo capsule at the same times.

Fifty-six of the subjects fulfilled the inclusion criteria and participated in the study, but four dropped out the study with these reasons: two due to dermatitis and one for digestive problems in the intervention group and one in the placebo group because of personal problems. Therefore, 52 participants [L-arginine (n = 25) and placebo (n = 27)] completed the trial ( Figure 1). Final statistical analyses were performed on the 52 participants. The rate of compliance in our study was high, such that approximately 100% of capsules were taken throughout the study in both groups. General characteristics of participants who received either L-arginine supplements or placebo are illustrated in Table 1. No significant differences were found in weight, BMI, physical activity, energy or protein intake between both groups ( Table 1). Because L-arginine levels in serum were not measured during the study, there was no possibility of getting correlation between arginine and age, BMI and PA.

**Table 1.** General baseline characteristics of athletic men who received either L-arginine supplements or placebo ^1^.

	L-arginine group ^3^ (n = 25)	Placebo group ^2^ (n = 27)	P value ^4^
Age (y)	21.32±4.59	20.40±4.04	0.44
BMI (kg/m ^2^)	23.34±4.02	24.01±4.53	0.574
Height (cm)	175.48±6.40	176±7.06	0.783
Weight (kg)	73.82±14.02	72.90±16.32	0.830
Physical activity (MET-minutes/week)	3941.44±987.70	3810.09±1199.69	0.670
Energy Intake (Kcal)	2251.46±362.01	2232.65±341.48	0.848
Protein Intake (g/d)	83.04±14.13	80.66±7.52	0.447

^1^ All values are means ±SDs

^2^ Received placebo 2000 mg per day during the study

^3^ Received L-arginine supplement 2000 mg per day during study

^4^ Obtained from independent-samples t test

The differences between the two groups in dietary intake during the trial are presented in Table 2. Dietary intake of energy, protein, carbohydrate, fat and arginine during study were not different between L-arginine and placebo groups.

**Table 2.** Dietary assessment in L-arginine and placebo groups.

Variables	L-arginine group n = 25	Placebo group n = 27	P-value †
Energy (Kcal)	2251.46±362.01	2232.65±341.48	0.848
Carbohydrate (g)	327.52±40.86	317.47±27.78	0.310
Protein (g)	83.04±14.13	80.66±7.52	0.459
Fat (g)	66.44±12.43	69.24±8.93	0.351
Arginine (mg)	388.73±464.33	437.97±458.29	0.702

Data is presented as mean and standard deviation

†Obtained from independent sample t test

**Table 3.** Fasting blood sugar, lipid profile, systolic and diastolic blood pressure in L-arginine and placebo groups.

Variables	L-arginine group (n = 25) ^1^				Placebo group (n = 27) ^2^				P-value ^4^
	Baseline	After	Change	P-value	Baseline	After	Change	P-value ^3^	
FBS (mg/dl)	99.28 ± 7.78	96.92 ± 7.86	-2.36 ± 2.04	0.001>	99.55 ± 5.65	99.89 ± 5.76	0.33 ± 1.39	0.223	0.001
TG (mg/dl)	74.48 ± 15.91	71 ± 15.67	-2.81 ± 3.01	0.001>	74.85 ± 14.39	74.92 ± 14.61	0.7 ± 2.11	0.857	0.001>
LDL (mg/dl)	100.08 ± 10.46	98.81 ± 10.91	-1.28 ± 1.65	0.01	98.55 ± 10.53	98.63 ± 10.78	0.07 ± 1.57	0.808	0.04
HDL (mg/dl)	59.31 ± 7.93	61.06 ± 7.16	1.04 ± 1.46	0.001>	60.11 ± 7.09	60.21 ± 6.85	0.08 ± 1.14	0.702	0.015
Cholesterol (mg/dl)	156.32 ± 17.53	153.96 ± 17.09	-2.36 ± 2.22	0.001>	158.92 ± 11.88	159.03 ± 11.74	0.11 ± 1.95	0.769	0.001>
SBP (mmHg)	115.41 ± 6.11	114.01 ± 4.78	-1.41 ± 3.07	0.032	107.22 ± 5.43	117.22 ± 4.66	0 ± 3.11	0.001	0.81
DBP (mmHg)	74.4 ± 6.56	73.01 ± 4.33	-1.41 ± 3.68	0.07	75.92 ± 4.16	75.18 ± 4.04	-0.74 ± 3.85	0.327	0.532

Data is presented as mean and standard deviation

Abbreviation: fasting blood sugar, triglyceride, low density lipoprotein, high density lipoprotein, systolic blood pressure, diastolic blood pressure

^1^ Received L-arginine 2000 mg per day during study

^2^ Received placebo 2000 mg per day during the study

^3^ Obtained from paired-samples t test

^4^ Obtained from independent-samples t test

Baseline and after intervention values of FBS, blood pressure and lipid profile are presented in ( Table 3). In this study, supplementation of 2000 mg L-arginine per day compared with placebo (2000 mg maltodextrin) for 45 days were given to participants. Levels of FBS, triglycerides, total cholesterol, LDL-c and HDL-c in L-arginine supplemented group compared with the placebo group showed statistically significant differences (P<0.05). The systolic and diastolic blood pressure before and after the intervention compared to the control group showed no significant difference (P>0.05) ( Table 3).

## Discussion

In this study, the effect of L-arginine supplementation on blood glucose, blood pressure and lipid profile in healthy male subjects between 18 and 35 years were examined. In the intervention group of subjects receiving 2000 mg daily of L-arginine pills for 45 days and the control group participants 2000 mg daily placebo pills (maltodextrin) consumed. The results showed that L-arginine supplementation significantly decreased the levels of FBS, triglycerides, LDL and cholesterol and a significant increase in HDL levels compared to the control group (P<0.05). However, there was no significant effect on systolic and diastolic blood pressure (P>0.05) ( Table 3).

In some studies, healthy individuals exhibited improved glucose metabolism after three to seven days of L-arginine supplementation ^19, 28^, a result that is in line with our study. Lucotti *et al.* demonstrated that L-arginine supplementation reduces blood sugar in patients with diabetes, which also parallels with our study, although our study was conducted on healthy people ^21^. There is evidence that long-term L-arginine intake can increase insulin sensitivity and improve the glycemic indices ^29^. It seems that an acute dose of L-arginine affects the levels of nitric oxide. In a study conducted by Natarajan *et al.*, supplementation with L-arginine improves glycemic sensitivity in patients with diabetes ^30^. In another study Mohamadian and colleagues demonstrated that nitric oxide precursors can improve blood glucose and glycosylated hemoglobin levels in Wistar rats with diabetes through antioxidant activity ^31^.

In a study conducted on people with type 2 diabetes, L-arginine supplementation reduced systolic and diastolic blood pressure, results that are inconsistent with our results ^32^. However, a study by Lerman *et al.*, found that L-arginine supplementation over 6 months had no significant effect on systolic and diastolic blood pressure in humans, which is in line with our study ^24^. Lekakis and colleagues found similar results where a daily 6 g oral dose of L-arginine did not have a significant effect on blood pressure of patients with essential hypertension ^23^.

Siani *et al.* lowering blood sugar, lowering blood pressure, increasing HDL, cholesterol and triglycerides and decreased following administration of L-arginine supplementation reported, that the results of this study agree with our study ^19^. In a study conducted in 2008 by Boger *et al.*, supplementation with L-arginine improved the function of the cardiovascular system in patients on hemodialysis ^32^. Several studies that were carried out on humans and male C57BL/6 mice have shown that L-arginine supplementation may be considered a new treatment for metabolic disorders and also has an effect of lowering blood pressure, adipose tissue and weight and improves insulin sensitivity ^33– 35^. In a study conducted by Martina *et al.* L-arginine supplementation of 2.1 g per day in combination with N-acetylcysteine at a dose of 2.1 g per day for 6 months has been shown to improve endothelial function ^36^.

In one study, M.A. Nascimento *et al.* showed that L-arginine supplementation in overweight men for 7 days reduced LDL and increased HDL, results that agree with the results of our study. However, unlike our findings, they did not see any effect on the levels of triglycerides and total cholesterol. These differing results may be due to the short duration of their study, as well as the high BMI and average age of their participants (46 ± 5) ^37^.

Mechanisms activated by L-arginine supplementation are still not fully understood. L-arginine can affect the molecular and cellular levels via complex mechanisms. Studies on multiple animal models and in a limited number of human subjects have shown that L-arginine can stimulate the development of brown adipose tissue mitochondria and induce the regulation of gene expression ^38^. Another study reported that L-arginine supplementation decreased blood pressure and total homocysteine levels ^39^. Indeed, nitric oxide is produced from arginine as an endothelium relaxation factor, which activates guanylyl cyclase. Guanylyl cyclase converted guanylyl triphosphate to cyclic guanylyl monophosphate which relaxes the smooth muscles that can cause a decrease in blood pressure ^40, 41^. Although the results of many studies are in line with our study, more are needed to determine the effects of differing L-arginine doses on CVD risk factors.

Some limitations of this study should be considered. First, we could not examine the effects of L-arginine supplementation on inflammation factors including tumor necrosis factor alpha (TNF-α) C-reactive protein (CRP) and interleukin-6 as CVD risk factors. Second, this study was conducted on males and it is not clear the effects of L-arginine supplementation on CVD risk factors on females. Third, in this study, we just enrolled healthy subjects and we did not examine the effects of L-arginine on patients with CVD.

## Conclusion

The intake of 2 g of L-arginine per day, for 45 days, could improve the lipid profile and FBS in male athletes, but has no effect on systolic and diastolic blood pressure. Further studies based on determination of L-arginine dose and with a larger sample size are needed to shed light on our findings.

## Data availability

The data referenced by this article are under copyright with the following copyright statement: Copyright: © 2017 Pahlavani N et al.

Data associated with the article are available under the terms of the Creative Commons Zero "No rights reserved" data waiver (CC0 1.0 Public domain dedication). 

Figshare: http://dx.doi.org/10.6084/m9.figshare.1265047 ^41^
