# Supplementary material for: L-arginine supplementation and risk factors of cardiovascular diseases in healthy men: a double-blind randomized clinical trial
Source: F1000Res. 2017 Jun 22;3:306. Originally published 2014 Dec 12. [Version 2] doi: 10.12688/f1000research.5877.2 (PMC5510020; doi:10.12688/f1000research.5877.2)
Supplement: Peer review history

# Peer review: Referee response for version 2

**DOI:** 10.5256/f1000research.12610.r23982

**Published:** 2017-07-03

**Authors:** Majid Ghayour-Mobarhan

**Affiliations:** 1 Cardiovascular Research Center, Mashhad University of Medical Science, Mashhad, Iran

The authors have edited the manuscript according to the referee comments.

I have read this submission. I believe that I have an appropriate level of expertise to confirm that it is of an acceptable scientific standard.

# Peer review: Referee response for version 1

**DOI:** 10.5256/f1000research.6279.r8320

**Published:** 2015-04-14

**Authors:** Majid Ghayour-Mobarhan

**Affiliations:** 1 Cardiovascular Research Center, Mashhad University of Medical Science, Mashhad, Iran

The title and abstract are appropriate, as are the methods and analysis. The conclusions presented are justified based on the results, and the data provided is sufficient.

Please make the following modifications in your article:

 

1. The statistical correlations between the arginine and the age, BMI and Physical activity should be assessed and reported.
2. On what basis was the arginine dosage selected?
3. The conclusion should be present in a separate section.

I have read this submission. I believe that I have an appropriate level of expertise to confirm that it is of an acceptable scientific standard, however I have significant reservations, as outlined above.

# Response

**Published:** 2017-05-16

**Authors:** Mostafa Jafari

**Affiliations:** Arak University of Medical Science, Iran

Dear Referee

 Your comments on my article with title (L-arginine supplementation and risk factors of cardiovascular diseases in healthy men: a double-blind randomized clinical trial)  were modified and submitted in new version.

 but in one comment  :(The statistical correlations between the arginine and the age, BMI and Physical activity should be assessed and reported)

 Because L-arginine levels in serum was not measured during the study, there was no possibility of getting correlation between arginine and age, BMI and PA

 Thanks

# Peer review: Referee response for version 1

**DOI:** 10.5256/f1000research.6279.r7756

**Published:** 2015-03-03

**Authors:** Roman Leischik

**Affiliations:** 1 Department of Cardiology, Witten/Herdecke University, Hagen, Germany

The study is well conducted. The data support the conclusions. We don`t know anything about the long-term results. For this issue it is necessary to perform long-term studies. L-Arginine supplement was used in sport, but its positive effects on it are not sure ^1^. Plasma nitrite levels were elevated compared to placebo during acute supplement in 5 km runners. We don`t know the long-term effects in patients with diabetes or patients with CAD over the years. But some cited studies in this well conducted study are promising. I think, because of the financial problems we don´t will have a well performed double blinded long-term study in the future. It is therefore quite beneficial to have a well conducted study, especially about possible adverse effects. It is interesting that lowering of blood pressure showed negative effects, but maybe the underlying mechanism in patients with metabolic syndrome might lead to other effects. If it is possible, it would be necessary to get a grant for a long-term study, because the adverse effects are low and possible positive effects might be significant.

The problem is that physical activity and healthy life style in general is more important as all supplements.  There is a great desire in patients for taking supplements or pills as a solution for their problems and diseases, because then there is no need for an individually effort of the patient itself. I think it is important to mention this. Further studies should be carried out comparing the effects of supplementation of L-Arginine and physical activity in patients. But we have to start somewhere with research and this study is one of the first well conducted and transparent studies.

It would be nice if the author can discuss the problem physical activity ^2^/life style ^3^ and supplements  in the discussion section. Socioeconomic differences might play a greater role as supplement with L-Arginine ^4^.

I have read this submission. I believe that I have an appropriate level of expertise to confirm that it is of an acceptable scientific standard.

## References

[1] SandbakkSBSandbakkOPeacockOJamesPWeldeBStokesKBohlkeNTjonnaAE: Effects of acute supplementation of L-arginine and nitrate on endurance and sprint performance in elite athletes. *Nitric Oxide*.2014; 10.1016/j.niox.2014.10.006 10.1016/j.niox.2014.10.006 Reference source 25445632

[2] Fiuza-LucesCGaratacheaNBergerNALuciaA: Exercise is the real polypill. *Physiology (Bethesda)*.2013;28(5) : 10.1152/physiol.00019.2013 330-358 10.1152/physiol.00019.2013 Reference source 23997192

[3] EdwardsonCLGorelyTDaviesMJGrayLJKhuntiKWilmotEGYatesTBiddleSJ: Association of sedentary behaviour with metabolic syndrome: a meta-analysis. *PLoS One*.2012;7(4) : 10.1371/journal.pone.0034916 10.1371/journal.pone.0034916 Reference source 22514690PMC3325927

[4] LindstromMHansonBSOstergrenPO: Socioeconomic differences in leisure-time physical activity: the role of social participation and social capital in shaping health related behaviour. *Soc Sci Med*.2001;52(3) :441-451 Reference source 1133077810.1016/s0277-9536(00)00153-2
